# Comparative analysis of chloroplast genomes in *Carica* species reveals evolutionary relationships of papaya and the development of efficient molecular markers

**DOI:** 10.3389/fpls.2025.1686914

**Published:** 2025-10-14

**Authors:** Yajin Chen, Xiaoxi Du, Lei Pan, Qiyuan Huang, Zhigang Hao

**Affiliations:** ^1^ Hainan Key Laboratory for Biosafety Monitoring and Molecular Breeding in Off-Season Reproduction Regions, Sanya Research Institute and Institute of Tropical Bioscience and Biotechnology, Chinese Academy of Tropical Agricultural Sciences, Sanya, Hainan, China; ^2^ School of Mathematics and Statistics, Qingdao University, Qingdao, China; ^3^ Hainan Seed Industry Laboratory, Sanya, Hainan, China; ^4^ Key Laboratory of Integrated Pest Management on Crops in Northwestern Oasis, Ministry of Agriculture and Rural Affairs, National Plant Protection Scientific Observation and Experiment Station of Korla, Xinjiang Key Laboratory of Agricultural Biosafety, Institute of Plant Protection, Xinjiang Uygur Autonomous Region Academy of Agricultural Sciences, Urumqi, China

**Keywords:** *Carica papaya* L., chloroplast genome, genetic diversity, molecular markers, evolutionary relationship

## Abstract

Papaya (*Carica papaya* L.) has a very high economic value, making it one of the “three major tropical herbaceous fruit trees” alongside banana and pineapple. However, it faces major challenges due to limited genetic diversity resulting from monocultures and barriers to interspecific hybridization, which seriously affect yield, quality, and disease resistance. To elucidate phylogenetic relationships among diverse cultivars and facilitate marker-assisted selection in breeding, we sequenced and analyzed the chloroplast genomes of 17 representative papaya cultivars, including commercial cultivars (e.g., ‘Guangmi’ and ‘Tainong’) and agricultural cultivars, by Illumina sequencing technology. The chloroplast genome showed a conserved tetrad-loop structure encoding 131 functional genes, and phylogenetic analyses using maximum likelihood (ML) revealed their evolutionary relationships and resolved two major clades. Whole-chloroplast genome sequence alignment analysis identified hypervariable non-coding regions, enabling the development of three high-resolution molecular markers (*trnG*–*trnR*, *rbcL*–*accD*, and *rpl12*–*rps19*). The three markers effectively distinguished key breeding lines (YZ1 and Tainong), providing a robust toolkit for germplasm innovation and marker-assisted breeding in papaya. This study not only provides a theoretical foundation and technical means for the genetic improvement of papaya but also serves as a reference for research on chloroplast resources in other tropical fruit trees.

## 1 Introduction

The genus *Carica*, a key economic group within the family Caricaceae (Avila-Hernandez et al., 2023), includes the model species *Carica papaya* L. (**Supplementary Figure S1**), renowned as the “king of fruits” due to its rich vitamin content (Leitao et al., 2022), proteolytic enzymes (Dominguez et al., 2006; Gomes et al., 2010), and medicinal properties (Haber et al., 2023). Although several species of the family Caricaceae were regionally cultivated, for example, *Vasconcellea pubescens* (Lemus-Mondaca et al., 2024), *C. papaya* is the only cultivated member of the genus *Carica*. The cross-incompatibility makes papaya highly vulnerable to pathogens and environmental stresses on account of its limited interspecies genetic diversity (Chávez-Pesqueira and Núñez-Farfán, 2017). Widely cultivated in tropical regions, papaya faces significant challenges in disease resistance breeding due to complex genetic backgrounds among species and cultivars, morphological plasticity, and frequent artificial hybridization leading to resistance gene introgression (Madronero et al., 2018; Mumo et al., 2020; Premchand et al., 2023). These factors complicate germplasm identification and hinder disease resistance breeding. While the nuclear genome of papaya has been extensively studied (Yue et al., 2022), research on its organellar genomes—particularly chloroplast (cpDNA)—remains limited (Lin et al., 2020).

Organellar genomes, originating from endosymbiotic events during early eukaryotic evolution, are critical for understanding plant evolution and adaptation (Hao et al., 2024a). The chloroplast genome, derived from cyanobacteria, typically exhibits a circular double-stranded structure (120–160 kb) with conserved gene arrangements, including photosynthesis-related genes (e.g., *psbA* and *rbcL*), transcription–translation machinery (e.g., rRNA and tRNA), and metabolic genes. Structural variations, such as expansions/contractions of inverted repeat (IR) regions, drive genome size diversity (Lin et al., 2024). The organellar genomes, owing to maternal inheritance (with rare exceptions), moderate evolutionary rates, and strong selective constraints on functional genes serve as invaluable tools for phylogenetic reconstruction, population genetics, and molecular marker development (Hao et al., 2024b). For example, novel molecular markers were developed by screening hypervariable regions from chloroplast genomes of four artificial *Taxodium* hybrids, accelerating breeding programs in this genus (Yue et al., 2023). Chloroplast genomics of seven *Panax* species enabled the development of 18 coding sequence (CDS)-derived species-specific Single Nucleic Polymorphism (SNP) markers for precise identification and cultivar authentication (Nguyen et al., 2020). Three hypervariable regions (*petA*–*psbJ*, *ndhF*–*rpl32*, and *ycf1*) were validated as candidate markers for distinguishing *Arnebiae Radix* and its substitutes (Wang et al., 2025). Molecular markers, including Amplified Fragment Length Polymorphism (AFLPs), Simple Sequence Repeats (SSRs), and Random Amplified Polymorphic DNA separately (RAPDs), and subsequently converted Sequence Characterized Amplified Region (SCARs), have been developed to differentiate the sex of *C. papaya* (Somsri et al., 1997; Deputy et al., 2002; Vashistha et al., 2016; Bui et al., 2024). To characterize the genetic diversity of species under the family Caricaceae, molecular markers were employed, which eventually resulted in the relocation of several members and the founding of the genus *Vasconcellea* (Kanchana-udomkan et al., 2014). The genetic polymorphism among different papaya varieties was revealed by a genome-wide SSR atlas (Vidal et al., 2014). Regional papaya cultivars in Sri Lanka were investigated for genetic diversity using SSR and Inter Simple Sequence Repeat (ISSR) markers (Warnakula et al., 2017). The practical molecular marker for distinguishing different cultivars remains unavailable. Chloroplast genomes, with their maternal inheritance, structural conservation, and balanced mutation rates, offer unique advantages for species identification and studies of adaptive evolution. Although recent advances highlight their role in identifying markers (Zheng et al., 2025), research on *Carica* cpDNAs remains scarce. These knowledge gaps impede the development of molecular markers for breeding.

This study aims to address the limitations of SSR and ISSR markers in discriminating different papaya cultivars by analyzing chloroplast genomes of 17 *Carica* varieties and cultivars. Our objectives are twofold: 1) to characterize the structural conservation and adaptive evolution of *Carica* cpDNAs and 2) to identify hypervariable regions associated with variety identification for marker development. The findings will facilitate marker-assisted selection, support intellectual property protection in breeding, and shed light on the evolutionary theories of *Carica* chloroplast genomes, bridging critical gaps between genomic insights and practical breeding applications.

## 2 Result

### 2.1 Assembly and annotation of chloroplast genomes

**Figure 1:**
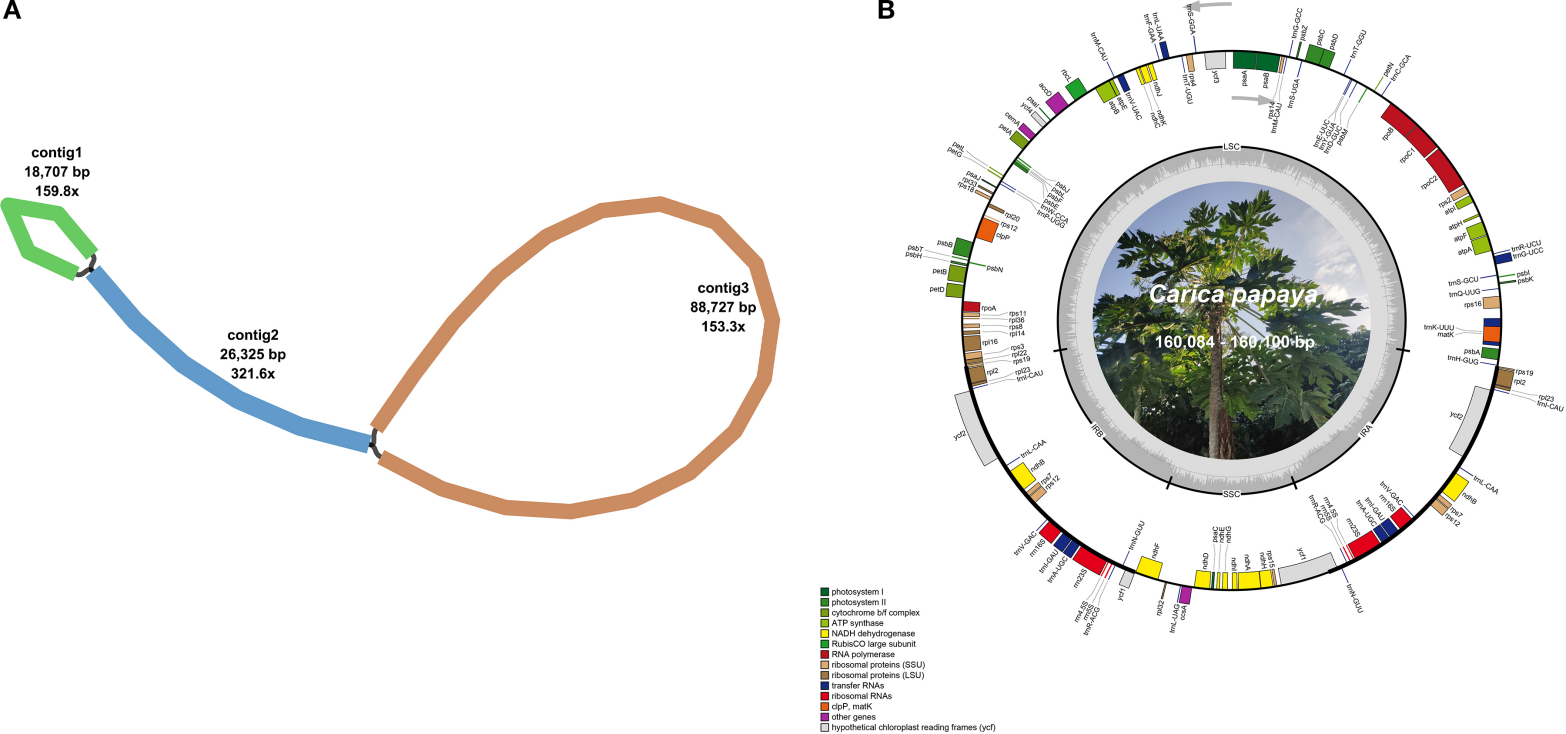
The graphic assembly and chloroplast genome maps of 17 *Carica* accessions. **(A)** The graphic plastomes consist of three contigs of different lengths, and they are connected to each other. The lengths of the three contigs are 18,707, 26,325, and 88,727 bp. Contig 2 is the repetitive sequence. **(B)** The genome map of the *Carica* plastomes. The figure shows the master circle of the *Carica* plastomes.

The Illumina HiSeq 2000 platform generated sequence data files totaling 4,963,041,000 to 6,473,551,500 bases per sequenced *C. papaya* genome. Through chloroplast genome assembly of sequencing data, we obtained circular chloroplast genomes for all 17 *Carica* species, as shown in **Figure 1**.

Based on sequence analysis, these papaya accessions were classified into two distinct types: Type 1 (160,084 bp; Guanine and Cytosine (GC) 36.89%), comprising eight accessions with identical sequences, and Type 2 (160,100 bp; GC 36.89%), represented by nine accessions exhibiting full sequence identity (**Table 1**). The chloroplast genomes of these 17 *Carica* accessions exhibited a conserved circular structure, annotated with 78 unique protein-coding genes (eight multi-copy), 29 tRNA genes (eight multi-copy), and four rRNA genes (all multi-copy). Protein-coding genes were classified into 15 functional families (**Table 2**). Although both chloroplast genome types exhibited conserved lengths and GC content within their respective groups, no differences were observed in gene content or copy numbers.

**Table 1:** Chloroplast genome architectures of two papaya types (*Carica papaya*).

Type	Sample name	Latin name	Total length (bp)	GC content (%)
Type 1	GDguangmi	*C. papaya*	160,084	36.89
	GDhongling1	*C. papaya*	160,084	36.89
	GDhongling2	*C. papaya*	160,084	36.89
	GXguire3hao1	*C. papaya*	160,084	36.89
	GXguire3hao2	*C. papaya*	160,084	36.89
	hongling5hao1	*C. papaya*	160,084	36.89
	hongling5hao2	*C. papaya*	160,084	36.89
	YZ1	*C. papaya*	160,084	36.89
Type 2	Daqing	*C. papaya*	160,100	36.89
	Hongfei1	*C. papaya*	160,100	36.89
	Hongfei2	*C. papaya*	160,100	36.89
	Tainong1	*C. papaya*	160,100	36.89
	Tainong2	*C. papaya*	160,100	36.89
	Tainong3	*C. papaya*	160,100	36.89
	Tainong4	*C. papaya*	160,100	36.89
	Tainong5	*C. papaya*	160,100	36.89
	Tainong6	*C. papaya*	160,100	36.89

**Table 2:** Gene composition in the chloroplast genome of *Carica papaya*.

Group of genes	Name of genes
Subunits of NADH-dehydrogenase	*ndhA*, *ndhB* (×2), *ndhC*, *ndhD*, *ndhE*, *ndhF*, *ndhG*, *ndhH*, *ndhI*, *ndhJ*, *ndhK*
Subunits of photosystem I	*psaA*, *psaB*, *psaC*, *psaI*, *psaJ*
Subunits of photosystem II	*psbA*, *psbB*, *psbC*, *psbD*, *psbE*, *psbF*, *psbH*, *psbI*, *psbJ*, *psbK*, *psbL*, *psbM*, *psbN*, *psbT*, *psbZ*, *ycf3*
Subunits of cytochrome *b*/*f* complex	*petA*, *petB*, *petD*, *petG*, *petL*, *petN*
Subunits of ATP synthase	*atpA*, *atpB*, *atpE*, *atpF*, *atpH*, *atpI*
Large subunit of rubisco	*rbcL*
Small subunit of ribosome	*rps2*, *rps3*, *rps4*, *rps7* (×2), *rps8*, *rps11*, *rps12* (×2), *rps14*, *rps15*, *rps16*, *rps18*, *rps19* (×2)
Large subunit of ribosome	*rpl2* (×2), *rpl14*, *rpl16*, *rpl20*, *rpl22*, *rpl23* (×2), *rpl32*, *rpl33*, *rpl36*
DNA-dependent RNA polymerase	*rpoA*, *rpoB*, *rpoC1*, *rpoC2*
rRNA genes	*rrn4.5S* (×2), *rrn5S* (×2), *rrn16S* (×2), *rrn23S* (×2)
tRNA genes	*trnA*-UGC (×2), *trnC*-GCA, *trnD*-GUC, *trnE*-UUC, *trnF*-GAA, *trnG*-GCC, *trnG*-UCC, *trnH*-GUG, *trnI*-CAU (×2), *trnI*-GAU (×2), *trnK*-UUU, *trnL*-CAA (×2), *trnL*-UAA, *trnL*-UAG, *trnM*-CAU (×2), *trnN*-GUU (×2), *trnP*-UGG, *trnQ*-UUG, *trnR*-ACG (×2), *rnR*-UCU, *trnS*-GCU, *trnS*-GGA, *trnS*-UGA, *trnT*-GGU, *trnT*-UGU, *trnV*-GAC (×2), *trnV*-UAC, *trnW*-CCA, *trnY*-GUA
Maturase	*matK*
C-type cytochrome synthesis gene	*ccsA*
Envelope membrane protein	*cemA*
Protease	*clpP*
Subunit of acetyl-CoA-carboxylase	*accD*
Genes of unknown functions Open Reading	*ycf1* (×2), *ycf2* (×2), *ycf4*

### 2.2 Codon usage bias

Codon usage bias analysis was performed on 78 unique protein-coding genes (PCGs) from the chloroplast genomes of two types of *C. papaya* varieties. The usage patterns of synonymous codons for each amino acid are detailed in **Supplementary Table S1**. Codon preference was assessed using the Relative Synonymous Codon Usage (RSCU) value. Codons with an RSCU value greater than 1.00 were considered preferentially used by their corresponding amino acid.

**Figure 2:**
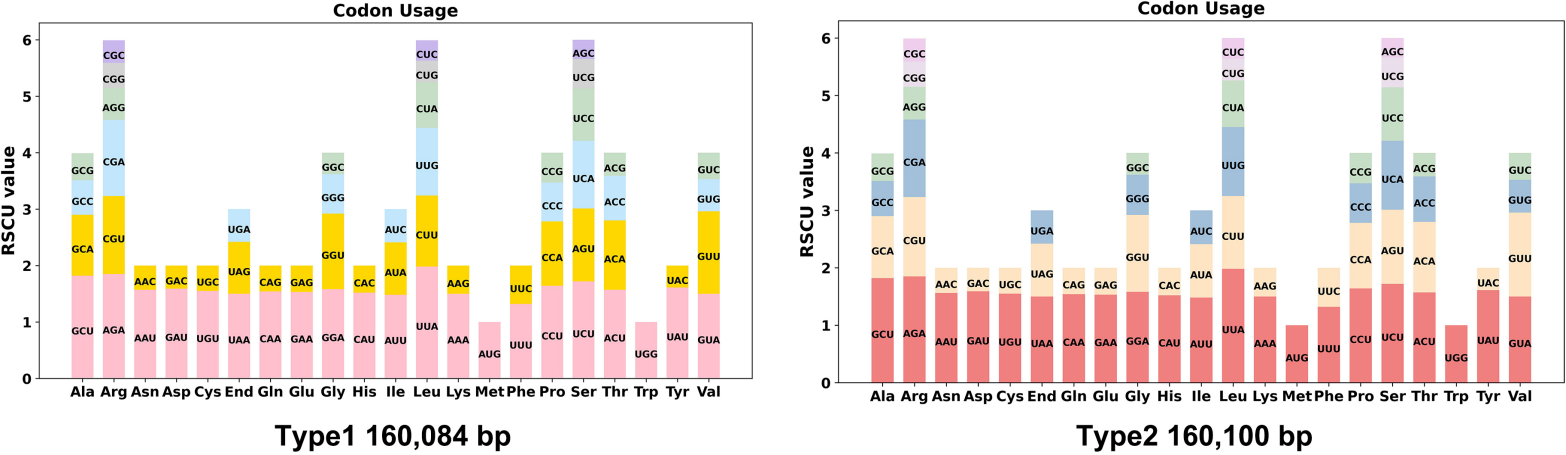
Codon preference analysis of two types of chloroplast genomes. Codon families are shown on the x-axis. RSCU values are the number of times a particular codon is observed relative to the number of times that codon would be expected for uniform synonymous codon usage. RSCU, Relative Synonymous Codon Usage.

As illustrated in **Figure 2**, the majority of chloroplast PCGs exhibited widespread codon usage bias. The notable exceptions were the start codon (AUG, encoding methionine) and the codon for tryptophan (UGG), both of which consistently showed an RSCU value of exactly 1.00. This is expected, as each has only a single synonymous codon. A striking example of strong codon preference was observed for the amino acid leucine (Leu). In both types of chloroplast genomes, Leu showed an extremely high preference for the codon UUA. This codon consistently achieved the highest RSCU value across all analyzed PCGs, reaching 1.98 in both chloroplast types. This represents a 5.2-fold preference over the least-used synonymous codons CUG and CUC (RSCU = 0.36–0.38), indicating strong selection for AT-rich codons compatible with the chloroplast's low GC content (36.89%). The identical bias pattern in both chloroplast types underscores the evolutionary conservation of translational optimization mechanisms.

### 2.3 Repeat sequence analysis

**Figure 3:**
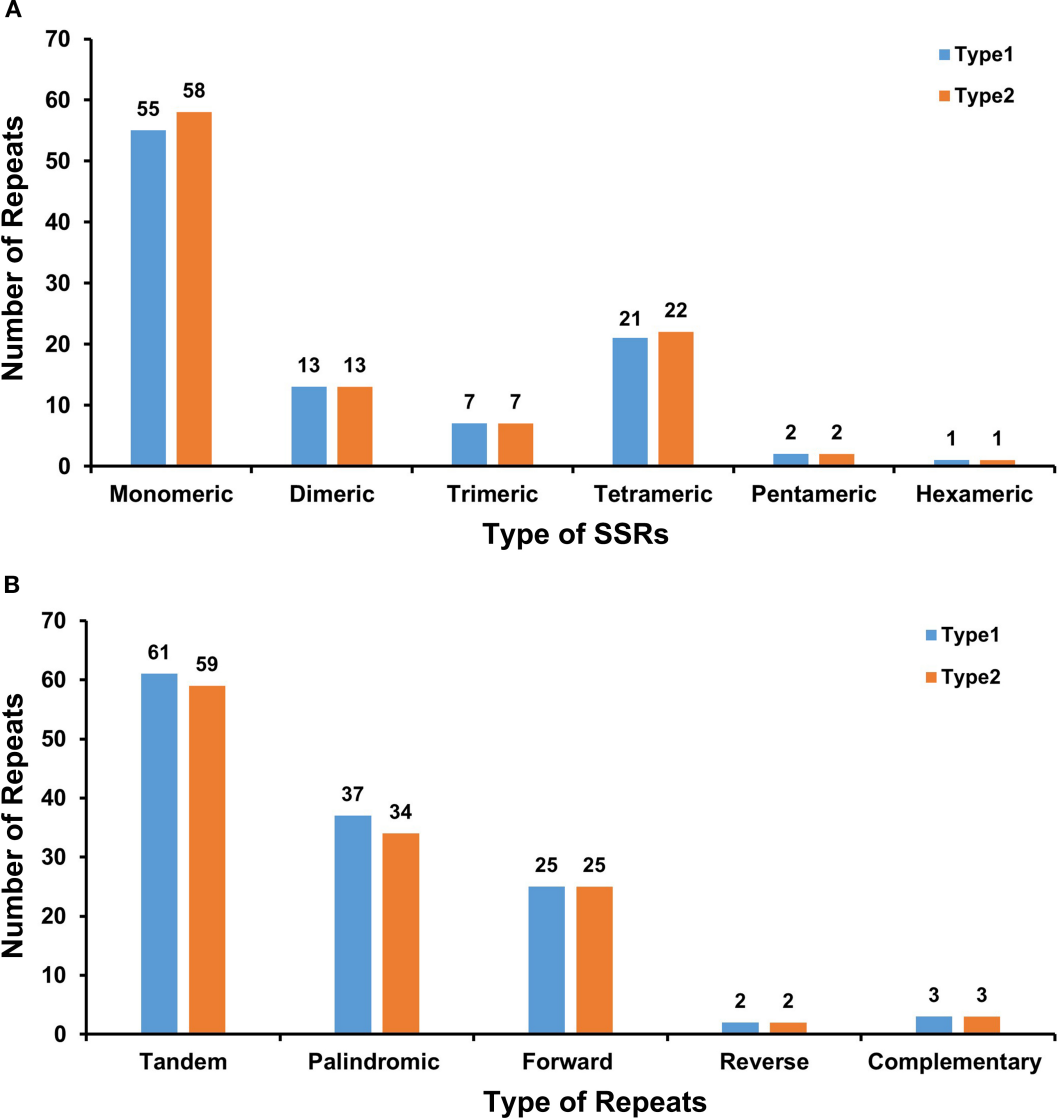
Comparative analysis of repeat sequences in two chloroplast genome types. **(A)** The number of SSRs of each type, with monomeric being the most and hexameric being the least. X-axis, SSR types; Y-axis, number of repeat units. Colors: Type 1 (blue), Type 2 (orange), and Type 3 (yellow). **(B)** The identified dispersed repeats (≥30 bp). X-axis, repeat sequence types; Y-axis, number of repeat elements. The green ribbons represent the forward repeats, the yellow ribbons represent the palindromic repeats, the red ribbons represent the complementary repeats, and the blue ribbons represent the reverse repeats. The detailed information about dispersed repeats can be found in **Supplementary Table S2**.

Comparative analysis revealed 99 and 103 SSRs in Type 1 and Type 2 chloroplast genomes, respectively. Both genomes exhibited similar distributions: monomeric and dimeric SSRs dominated (68.69% in Type 1 and 68.93% in Type 2), with thymine (T) monomers being the most prevalent (54.55% of Type 1 monomers and 56.90% in Type 2). Only one hexameric SSR was detected per genome. Tandem repeat analysis identified 61 repeats in Type 1 and 59 in Type 2, confirming conserved genomic architecture between types (**Figure 3A**, **Supplementary Table S2**).

Dispersed repeats ≥30 bp showed parallel patterns: Type 1 contained 67 repeat pairs (37 palindromic, 25 forward, 2 reverse, and 3 complementary), while Type 2 had 64 pairs (34 palindromic, 25 forward, 2 reverse, and 3 complementary). Both genomes shared identical maximum lengths for key repeats—26,325 bp for palindromic and 60 bp for forward repeats—indicating structural conservation at repeat boundaries (**Figure 3B**, **Supplementary Tables S3**, **S4**).

### 2.4 Phylogenetic analysis

**Figure 4:**
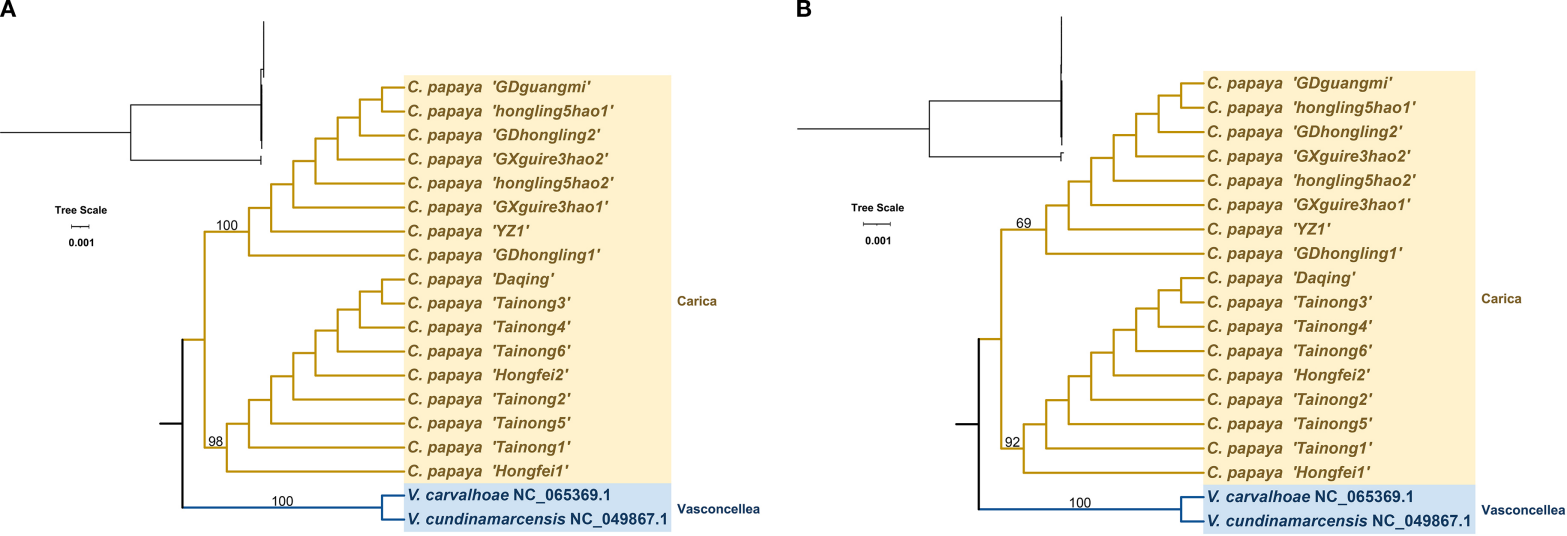
Phylogenetic trees of Carica papaya cultivars and related species based on complete chloroplast genome sequences **(A)** and conserved protein-coding sequences (CDS) **(B)**. Vasconcelles species were used as outgroups.

A maximum likelihood phylogenetic tree was constructed based on the complete chloroplast genomes and 78 shared protein-coding genes using two *Vasconcellea* species (*Vasconcellea carvalhoae* and *Vasconcellea cundinamarcensis*) as outgroups. The resulting topology, presented in **Figure 4**, is consistent with the Angiosperm Phylogeny Group (APG) classification and clearly resolves two major clades. However, the topology of the ITS tree showed incongruence with the chloroplast tree (**Supplementary Figure S3**).

Type 1 includes eight accessions (GDguangmi, GDhongling1, GDhongling2, GXguire3hao1, GXguire3hao2, hongling5hao1, hongling5hao2, and YZ1) with identical chloroplast genomes. These genomes are 160,084 bp in length and have a GC content of 36.89%. Type 2 comprises nine accessions (Daqing, Hongfei1, Hongfei2, Tainong1, Tainong2, Tainong3, Tainong4, Tainong5, and Tainong6), which also share identical chloroplast sequences. These genomes are slightly longer at 160,100 bp, with the same GC content of 36.89%.

### 2.5 IR region expansion/contraction analysis

**Figure 5:**
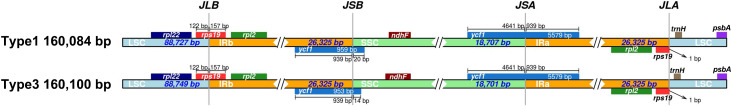
LSC/SSC/IR junction comparisons across two papaya types. Genes located near the boundaries are shown above or below the main line. JLB, JSB, JSA, and JLA denote the junctions of LSC/IRb, IRb/SSC, SSC/IRa, and IRa/LSC, respectively. JLB, junction of LSC/IRb (large single copy/inverted repeat b). JSB, junction of IRb/SSC (inverted repeat b/small single copy). JSA, junction of SSC/IRa (small single copy/inverted repeat a). JLA, junction of IRa/LSC (inverted repeat a/large single copy). LSC, large single-copy region. SSC, small single-copy region. Ira and IRb, inverted repeat regions a and b (the two copies of the repeat sequence in the chloroplast genome).

The chloroplast genomes of *Carica* varieties exhibited four IR–Single Copy (SC) boundaries (LSC-IRb, IRb-SSC, SSC-IRa, and IRa-LSC). Comparative analysis identified seven genes (*rpl22*, *rps19*, *rpl2*, *ycf1*, *ndhF*, *trnH*, and *psbA*) spanning or adjacent to IR–SC boundaries. Interestingly, *rps19* and *ycf1* showed conserved boundary-spanning structures across all 17 varieties. While IR lengths remained invariant (26,325 bp), minor variations in large single-copy (LSC)/small single-copy (SSC) lengths were observed among cultivars (**Figure 5**, **Table 3**). The 16-bp expansion of the IR region in Type 2 chloroplast genomes specifically resulted from *ycf1* extension into IRb (penetrating 15 bp deeper than in Type 1), while *rps19* uniformly expanded 8 bp into the LSC across all varieties. Critically, all cultivars within Type 1 (n = 8) and Type 2 (n = 9) exhibited identical IR boundaries, confirming the strict evolutionary conservation of inverted repeat architecture within each lineage.

**Table 3:** Genomic characteristics of two papaya chloroplast types.

Type	Region	Start site	Stop site	Length (bp)	GC content (%)
Type 1	LSC	1	88727	88,727	34.70
	IR	88728	115052	26,325	42.68
	SSC	115053	133759	18,707	31.01
	IR	133760	160084	26,325	42.68
Type 2	LSC	1	88749	88,749	34.69
	IR	88750	115074	26,325	42.68
	SSC	115075	133775	18,701	31.03
	IR	133776	160100	26,325	42.68

LSC, large single copy; IR, inverted repeat; SSC, small single copy.

### 2.6 Nucleotide diversity (Pi) analysis

Sliding window analysis (window, 400 bp; step, 200 bp) using DnaSP v6.0 identified four hypervariable regions (*ccsA*–*ndhD*, *ndhD*, *psbC*, and *ycf1*; Pi > 0.002), highlighting the overall conservatism of *Carica* chloroplast genomes. LSC and SSC regions exhibited higher Pi values than IRs.

Genome-wide nucleotide diversity analysis across 17 *Carica* chloroplast genomes revealed pronounced regional heterogeneity in polymorphism distribution. The IR regions exhibited absolute conservation, while the LSC region showed moderate variation. Strikingly, the SSC compartment demonstrated substantial diversity. Four hypervariable regions (*ccsA*–*ndhD*, *ndhD*, *psaC*, and *ycf1*; Pi > 0.002) were exclusively localized within the SSC region.

**Figure 6:**
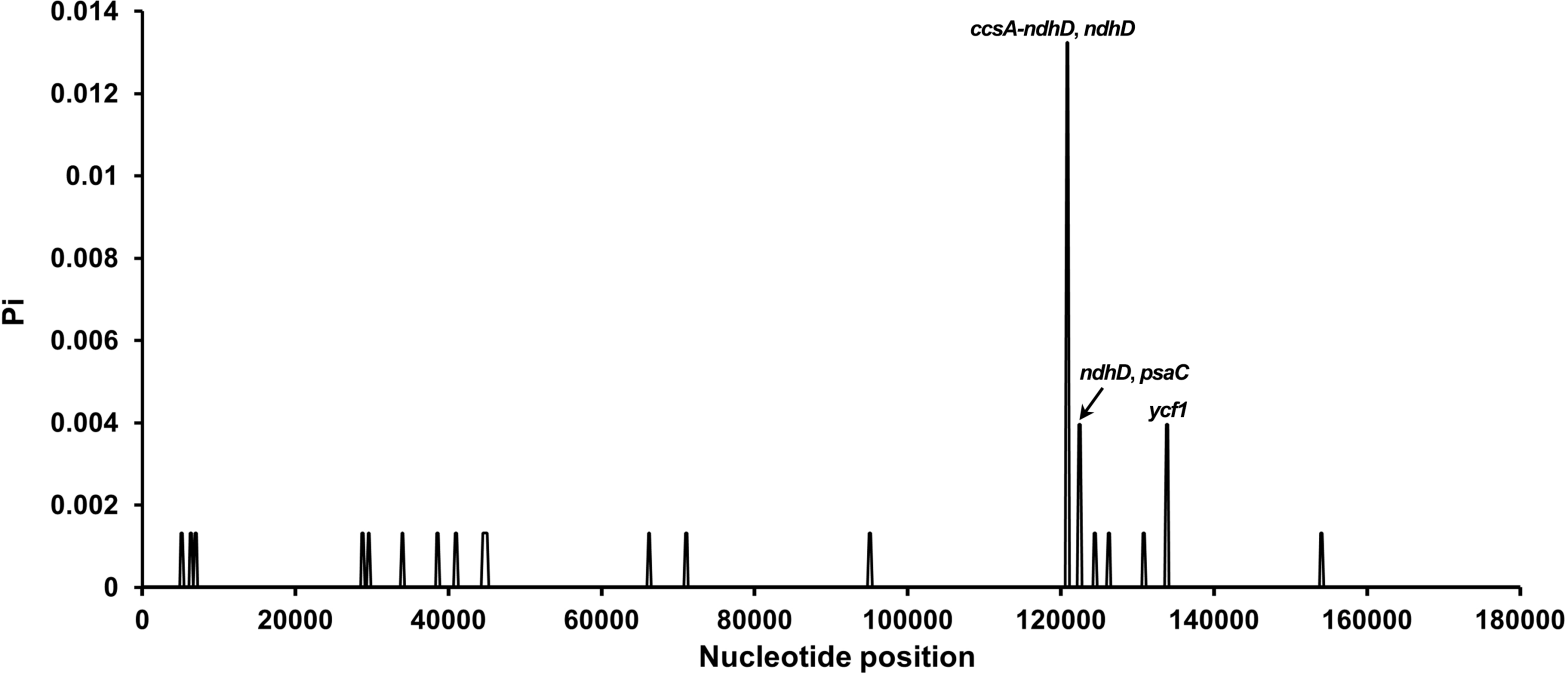
Sliding window analysis of nucleotide diversity (Pi) in 17 *Carica* chloroplast genomes. Each point represents nucleotide diversity calculated per 400-bp window. Peaks of diversity are annotated with corresponding gene names: *ccsA*–*ndhD* and *ndhD*, *ndhD* and *psaC*, and *ycf1*.

Among the identified hypervariable zones, the *ccsA*–*ndhD* intergenic spacer (IGS) displayed peak polymorphism (Pi = 0.01324), representing the most divergent non-coding region. Protein-coding genes *ycf1*, *psaC*, and *ndhD* (Pi = 0.00397) contained significant genic variations. These regions collectively constitute the primary mutation hotspots driving interspecific differentiation. mVISTA alignment confirmed greater sequence divergence in non-coding regions, with IRs showing >99% similarity (**Figure 6**, **Supplementary Table S5**).

### 2.7 Synteny analysis

**Figure 7:**
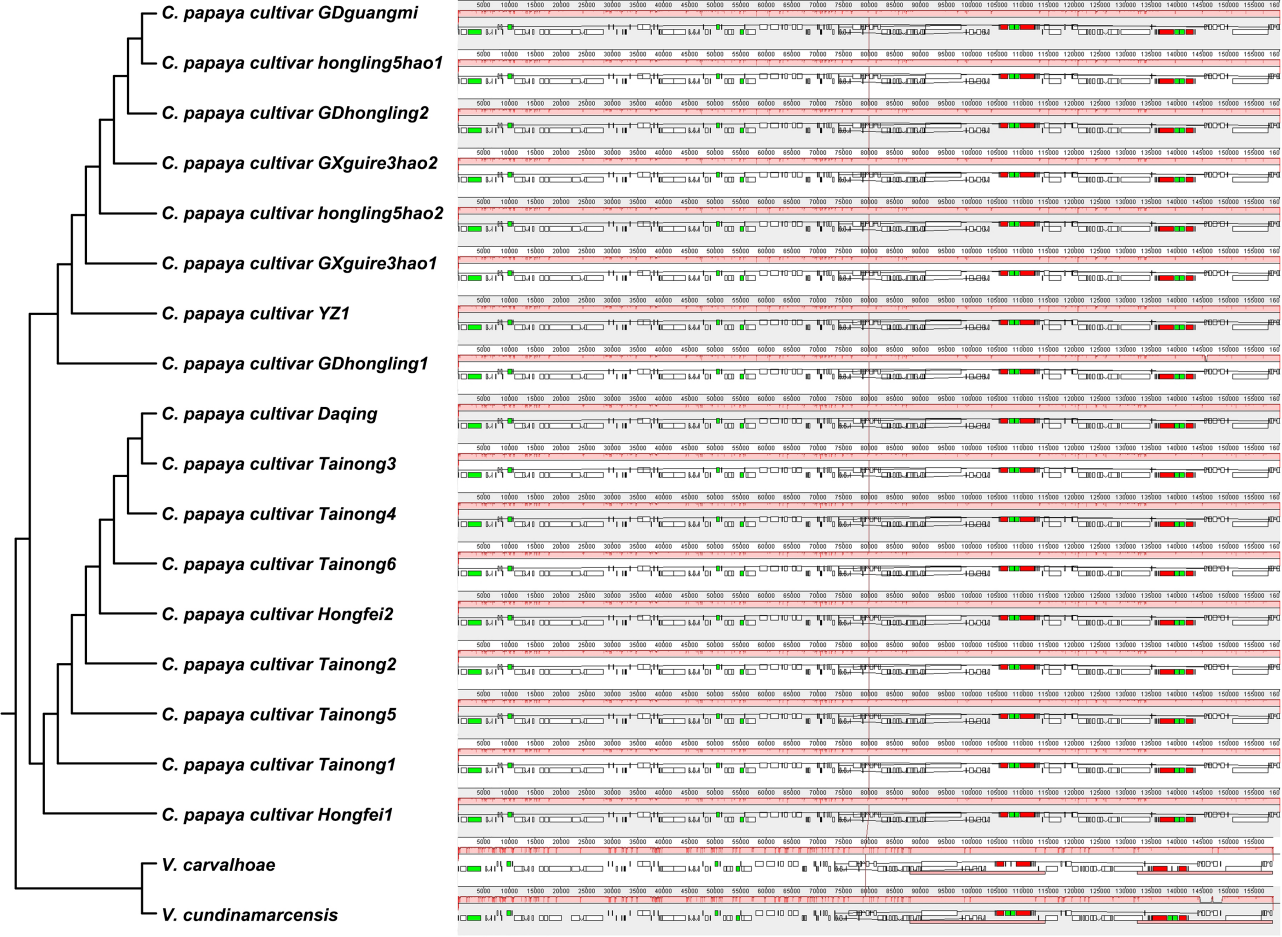
Whole-genome alignments of two chloroplast types (Mauve). The colorful bars indicate the mitogenomes, and the ribbons show the homologous sequences between the adjacent varieties. The blue ribbons indicate regions with homology, and the red ribbons indicate where the inversion occurred. The homologous blocks less than 0.5 kb in length are not retained, and regions that fail to have a homologous block indicate that they are unique to the varieties.

Whole-genome alignments using Mauve v2.4.0 demonstrated near-perfect structural conservation across 17 *Carica* chloroplast genomes, with identical gene order preserved in all LSC, IR, and SSC regions and no detectable inversions, translocations, or large InDels. The sole structural variation was a 16-bp Type 2-specific expansion at the IRb/SSC boundary. Phylogenetic congruence between synteny patterns and maximum likelihood trees underscores structural stability as a hallmark of chloroplast evolution in Caricaceae, with micro-variations at junctional regions providing molecular footprints of domestication history (**Figure 7**, **Supplementary Figure S3**).

### 2.8 Molecular marker development

**Figure 8:**
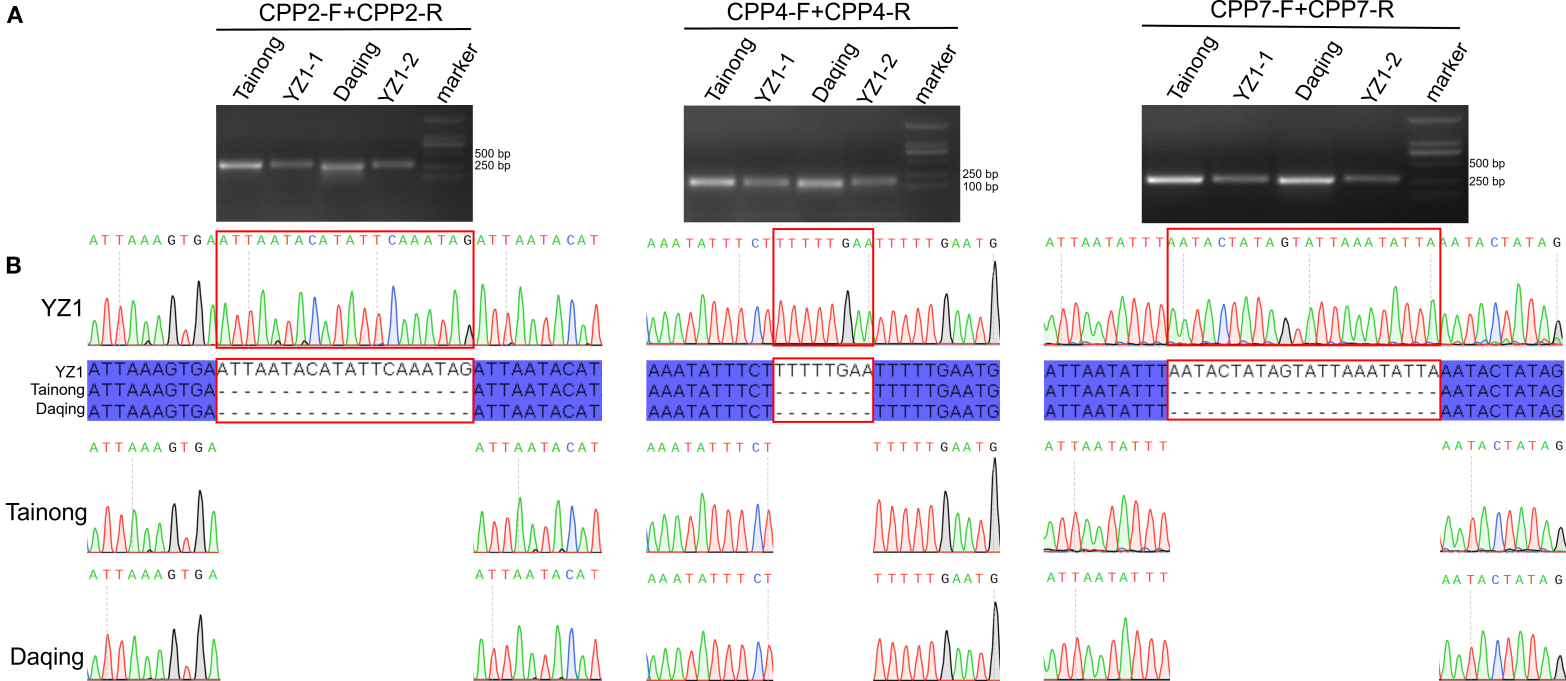
The alignment of the molecular markers of the PCR products amplified using the primers. **(A)** Barcode verification of four papaya varieties by PCR amplification using selected primer pairs. Marker lane was the DL2000 marker. **(B)** Sanger sequencing chromatogram of specific PCR product identified by gel electrophoresis.

Hypervariable non-coding regions (*trnG*–*trnR*, *rbcL*–*accD*, and *rpl12*–*rps19*) were prioritized for marker design. Three primer pairs were synthesized based on the left and right flanks of InDels and tested by PCR amplification on YZ1, Tainong, and Daqing (**Supplementary Table S6**). Specific fragments were successfully amplified by the following primer pairs: CPP-2F + CPP-2R and CPP-4F + CPP-4R. As expected, the sizes of specific PCR products were between 100 and 500 bp (**Figure 8A**). Three InDels discovered above were confirmed by Sanger sequencing of the PCR product, enabling the discrimination of hybrid cultivars undetectable by morphology (**Figure 8B**).

## 3 Discussion

### 3.1 The conservative papaya chloroplast genome harbors evolutionary drivers for disease resistance

Since the chloroplast genome is critical to papaya biology, the papaya chloroplast genome was investigated through high-throughput genome sequencing with experimental validation in 17 varieties. In earlier studies, attempts were made for chloroplast genome assembly by mostly undertaking next-generation sequencing (Lin et al., 2024). The main purpose of this work was to decipher the chloroplast genomes of multiple commercial cultivars and traditional landraces of papaya (*C. papaya*). As mentioned in the Introduction, chloroplast genomes are highly conserved across plant species (Hao et al., 2024a). This study reveals a high degree of conservation in *Carica* chloroplast genomes, characterized by stable gene synteny, invariant IR lengths (26,325 bp across all 19 varieties), and low nucleotide diversity (only four regions with Pi > 0.002). However, dynamic IR boundary shifts, as evidenced by the transboundary distribution of *ycf1* and *rps19*. Ycf1 (Tic214) was previously reported as a transmembrane subunit of the chloroplast TIC (translocon at the inner chloroplast membrane) complex, which transports cytosolic proteins into the chloroplast and collaborates with TOC (translocon at the outer chloroplast membrane) (Liu et al., 2023). Given the fundamental position of chloroplasts in plant immunity, the successful widespread cultivation of papaya in tropical regions implies that *ycf1* may play roles in adaptability by regulating defense and stress responses, such as reactive oxygen species, Ca^2+^, salicylic acid, and jasmonic acid (Xing et al., 2025; Liu et al., 2024).

### 3.2 Efficacy of molecular markers and their potential in breeding

The *rbcL*–*accD*, *rpl12*–*rps19*, and *trnG*–*trnR* marker combinations achieved 100% accuracy in distinguishing disease-resistant and susceptible cultivars, especially those among the Tainong clade and YZ1 clade, demonstrating the utility of chloroplast markers in papaya germplasm screening. However, the overall low genetic diversity of papaya chloroplast genomes may arise from genetic bottlenecks during domestication or convergent selection for environmental adaptation. To explore the linkage of the markers to the nuclear genome, the ITS region was analyzed and found extremely conserved, making it unsuitable for developing molecular markers among these cultivars (**Supplementary Figure S3**). Interestingly, hypervariable regions (*ycf1*) located in LSC/SSC regions (Pi > IRs) highlight the role of non-coding sequence variation in adaptive divergence. While these markers are effective, sole reliance on chloroplast data may inadequately resolve complex agronomic trait networks (Huang et al., 2024; Wang et al., 2024). Integration with nuclear loci through multi-omics approaches is critical for comprehensive profiling.

The presence of such phylogenetic topology is a unique finding. We report a novel phylogenetic topology revealing unexpected evolutionary relationships in *C. papaya*. Our results provide compelling evidence through Illumina sequencing and experiments. The molecular markers presented here are a cost-effective detection protocol. Phylogenetic congruence with APG classifications supports the monophyly of *Carica*, yet limited interspecific divergence may reflect recent domestication or gene flow driven by artificial selection for resistance traits. Unfortunately, no correlation of agronomic traits and geographical origins of cultivars among the two clades and the molecular markers was found. Further work focusing on the mechanisms shaping important agronomic traits, including fruit size, fruit color, and papaya ringspot virus (PRSV) resistance, using Genome-Wide Association Studies (GWAS) on different cultivars and Quantitative Trait Locus (QTL) on Recombinant Inbred Line (RILs) is needed.

### 3.3 Limitations and gaps in genetic diversity resolution

Our results are encouraging and should be validated in a large variety. The focus on cultivated varieties, with limited inclusion of wild relatives, likely underestimates natural genetic diversity within the genus. Although analyses of the papaya chloroplast genome are extensively documented, research on mitochondria-associated information remains limited. Analyses restricted to chloroplast genomes preclude insights into nuclear–mitochondrial interactions influencing disease resistance. The hypothesized link between *ycf1* and environmental adaptation remains untested via functional assays (e.g., gene knockout or overexpression), limiting our mechanistic understanding.

### 3.4 Translating genomic insights into applications

An important question for future work is to focus on the following areas: combining chloroplast, mitochondrial, and nuclear genomes to construct multi-layered phylogenetic networks, elucidating horizontal gene transfer; employing CRISPR/Cas9 to create *ycf1* mutants and validate its role in environmental adaptation; and developing hybrid marker panels (chloroplast-nuclear) to enhance resolution in cultivar identification and breeding.

Achieving durable and broad-spectrum resistance to multiple pathogens is continually being pursued in crop breeding (Li et al., 2020; Ning and Wang, 2018). PRSV and papaya leaf-distortion mosaic virus (PLDMV) are devastating pathogens in papaya cultivation and could lead to over 60% yield loss (Kung et al., 2009; Maoka and Hataya, 2005). Natural resistance to PRSV exists in *Carica cauliflora*, *Carica pubescens*, and *Carica quercifolia*. However, cultivated species *C. papaya* exhibits hybrid incompatibility with the wild species, making it difficult to introduce PRSV resistance through hybrid breeding. Species-specific PRSV resistance has been achieved by introducing conserved target sequences of *CP*, *NIb*, and *Hc-Pro* into cultivated papayas (Ferreira et al., 2002). Due to the genetic instability of RNA viruses, target sequence-dependent RNAi-induced PRSV resistance is highly prone to loss (Zhao et al., 2015; Tuo et al., 2013). The lack of PLDMV resistance in cultivated papayas has led to the prevalence of the virus during recent years (Zhao et al., 2016). Discovering and incorporating PRSV/PLDMV resistance to create elite cultivars will be highly desirable (Hu et al., 2023). The molecular markers developed in this study realized the marker-assisted selection and aggregation of resistance to different pathogens in cultivated papayas.

Additionally, exploring chloroplast-encoded viral response elements (e.g., non-coding RNAs) could identify novel targets for disease resistance. While this study establishes a framework for chloroplast-driven breeding in tropical crops, interdisciplinary collaboration is essential to translate genomic discoveries into practical agricultural solutions.

## 4 Methods

### 4.1 Plant materials and DNA preparation

*C. papaya* plants used in this study were obtained from farmlands in Yazhou District, Sanya, China, and the germplasm resources nursery maintained by the Chinese Academy of Tropical Agricultural Sciences (near 18°23′21.4″N, 109°09′51.5″E). Chloroplast DNA was extracted from fresh leaf tissue and used to construct 350-bp paired-end libraries, which were sequenced on the Illumina NovaSeq 6000 platform (PE150 strategy) (Verstraeten et al., 2025). DNA was extracted using Cetyltrimethylammonium bromide (CTAB) and trichloromethane:isopentanol, precipitated by isopropanol, rinsed with 75% ethanol, and dissolved in distilled water. The same leaf tissue was stored in a freezer at −80 °C. Seeds were collected and preserved for germplasm at 16 °C.

### 4.2 Genome sequencing, assembly, and annotation

Raw reads were used for the assembly of chloroplast genomes using GetOrganelle v1.7.5 with default parameters (Jin et al., 2020). Genome annotation was performed using CPGAVAS2 (Shi et al., 2019), followed by manual correction of annotation errors in Apollo v3.0 (Lewis et al., 2002). The tRNA and rRNA genes were predicted using tRNAscan-SE (Chan et al., 2021) and BLASTn (Chen et al., 2015), respectively. Chloroplast genome maps were visualized using CPGView and OGDRAW (Greiner et al., 2019; Liu et al., 2023).

### 4.3 Nucleotide diversity analysis, phylogenetic analysis, and IR boundary dynamics

Genome sequences were aligned with MAFFT v7 (Katoh and Standley, 2013), and nucleotide diversity (Pi) was calculated using DnaSP v6.0 (Rozas et al., 2017) with a sliding window approach (window, 500 bp; step, 100 bp). Sequence similarity across chloroplast genomes was assessed using mVISTA in ShuffleLAGAN mode (Frazer et al., 2004). Multiple alignment and phylogenetic analysis were conducted and visualized using MEGA 12, iTOL, IQ-TREE and PhyloSuite (Kumar et al., 2024; Letunic and Bork, 2024; Minh et al., 2020; Zhang et al., 2020). IRscope was used for visualizing LSC, SSC, and IR boundaries (Amiryousefi et al., 2018). Whole-genome alignments of chloroplast sequences were performed using Mauve to detect structural rearrangements and assess synteny (Darling et al., 2004). ITS2 sequences were obtained by processing raw sequencing data using ITSxpress for region extraction (Einarsson and Rivers, 2024; Rivers et al., 2018), followed by VSEARCH for merging, quality filtering, and dereplication (Rognes et al., 2016).

### 4.4 Molecular marker development

Primer design was conducted using NCBI Primer-BLAST (Ye et al., 2012). Primers were synthesized by BioSune Biotechnology Co., Ltd., Chunshen Rd., Minhang District, Shanghai, China. PCR amplification was performed using 2 × Rapid Taq Plus Master Mix (Dye Plus) purchased from Nanjing Vazyme Biotech Co., Ltd., Kechuang Rd., Nanjing, China, optimized via temperature gradient screening for annealing temperature, and extending at 72 °C for 1 min following the user manual. PCR products were separated by 1.5% (w/v) agarose gel electrophoresis at 120 V. Specific bands were excised, purified, and subsequently validated through Sanger sequencing using a 3730xl DNA Analyzer by Beijing Tsingke Biotech Co., Ltd., 3 Jinghai Rd., Daxing District, Beijing, China. Primer sequences are listed in **Supplementary Table 1**.

## Data Availability

The datasets presented in this study can be found in online repositories. The names of the repository/repositories and accession number(s) can be found in the article/**Supplementary Material**.
