# Supplementary material for: Comparative analysis of chloroplast genomes in Carica species reveals evolutionary relationships of papaya and the development of efficient molecular markers
Source: Front Plant Sci. 2025 Oct 14;16:1686914. doi: 10.3389/fpls.2025.1686914 (PMC12560180; doi:10.3389/fpls.2025.1686914)
Supplement: Supplementary file 1 [file Table1.docx]

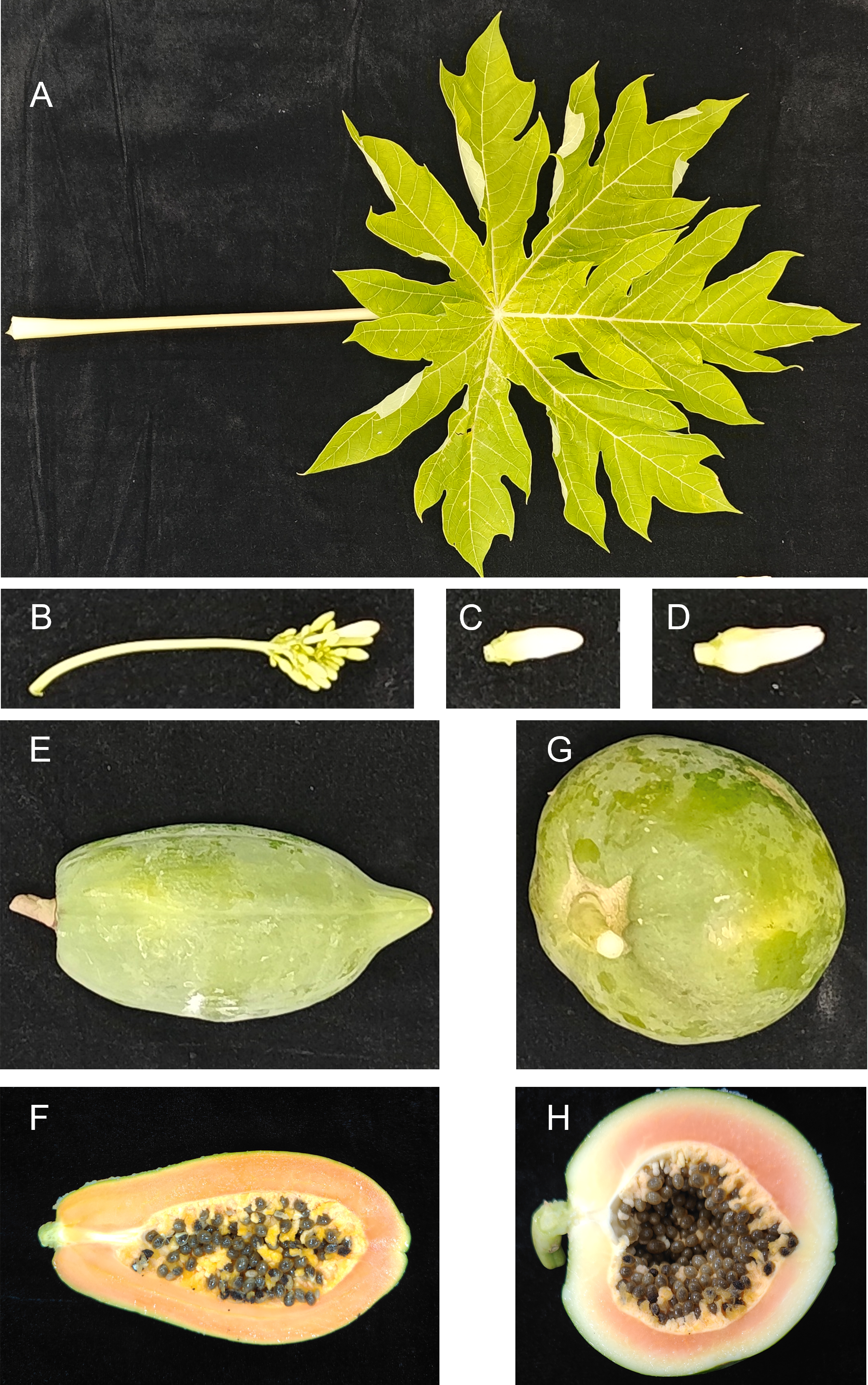


Figure S1 Morphology of *Carica papaya*.

A. Leaf. B. Male flowers. C. Hermaphrodite flower. D. Female flower. E, F. Hermaphrodite fruit. G, H. Female fruit.


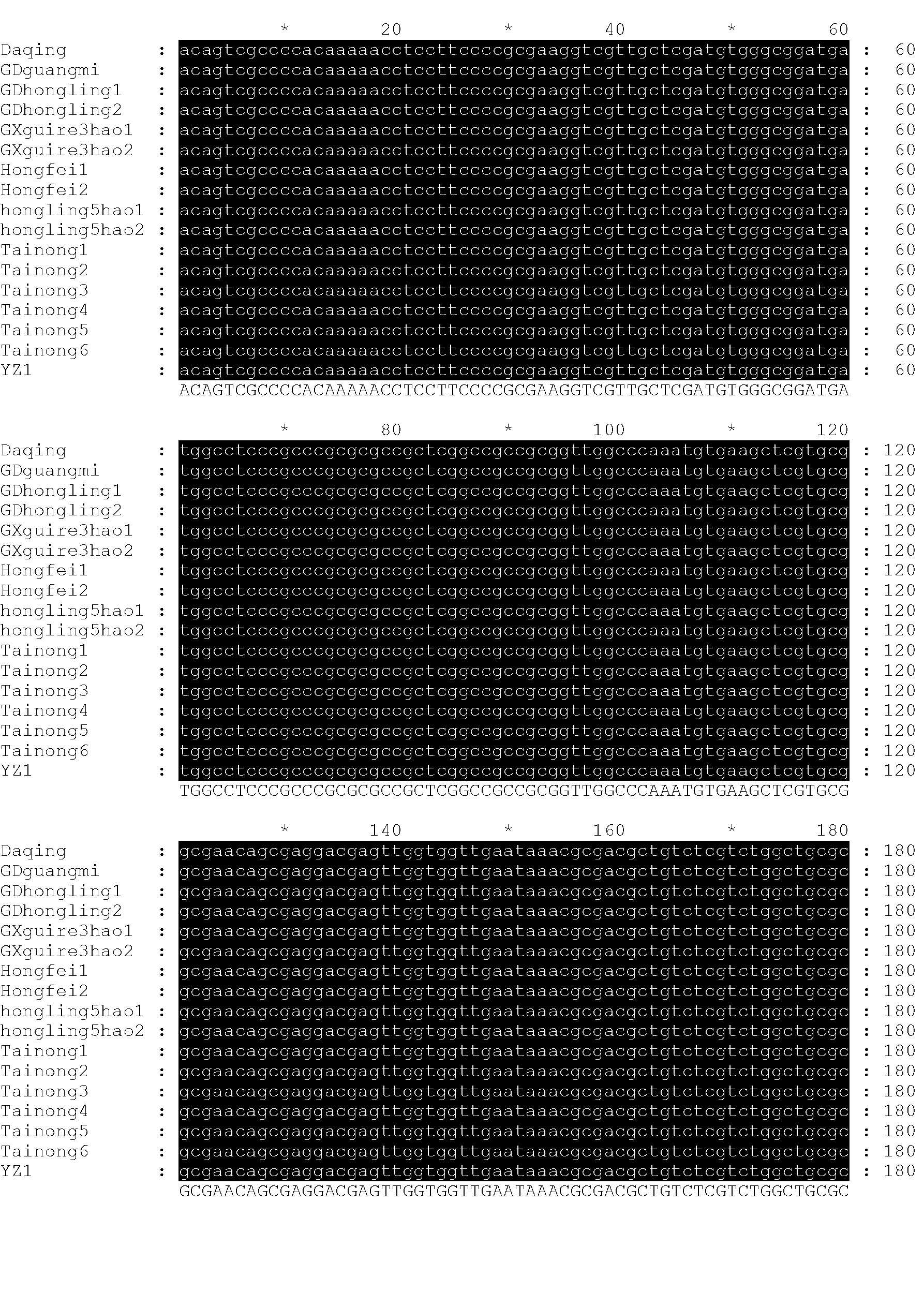


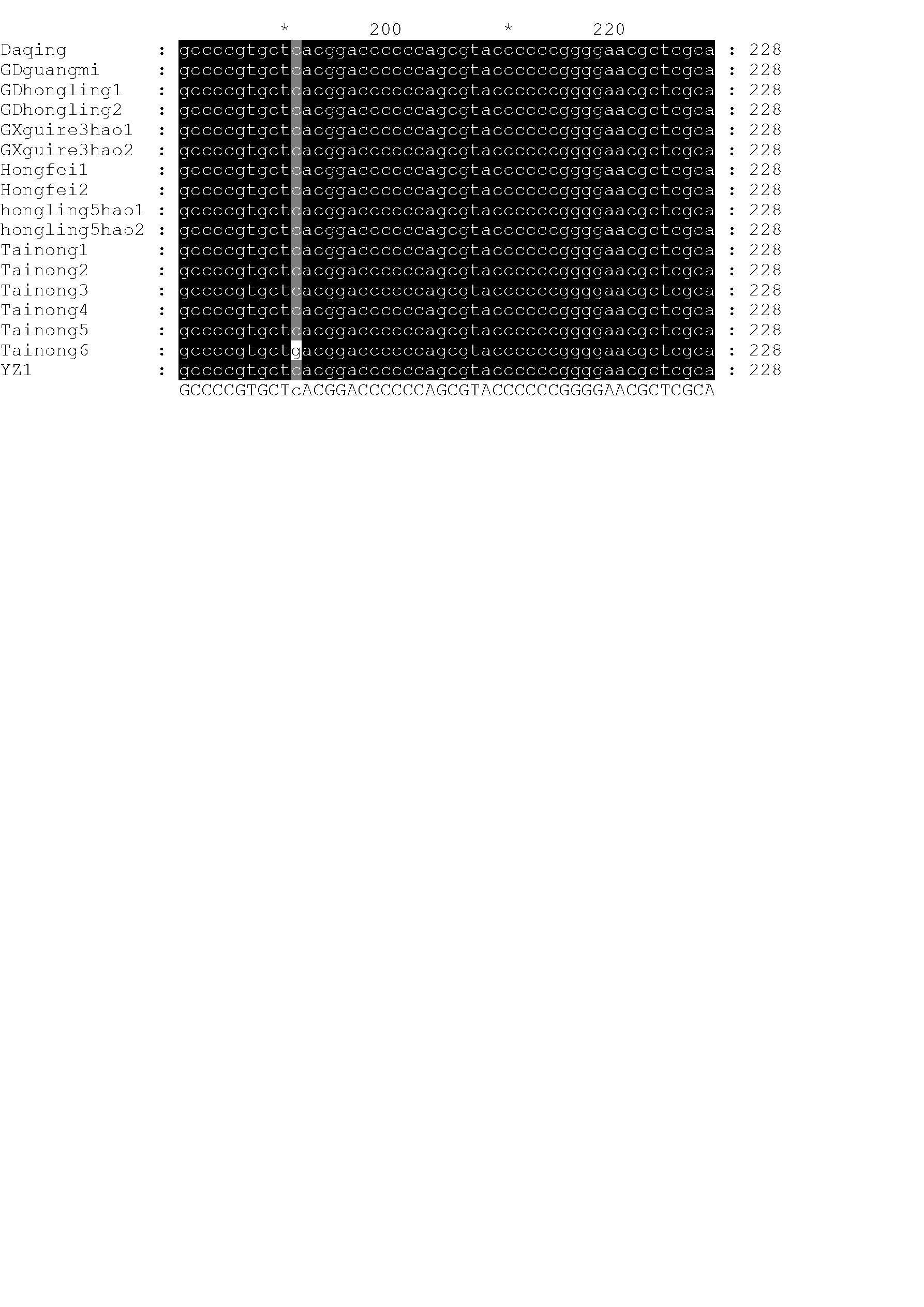


Figure S2 ITS sequences of 17 *Carica* cultivars


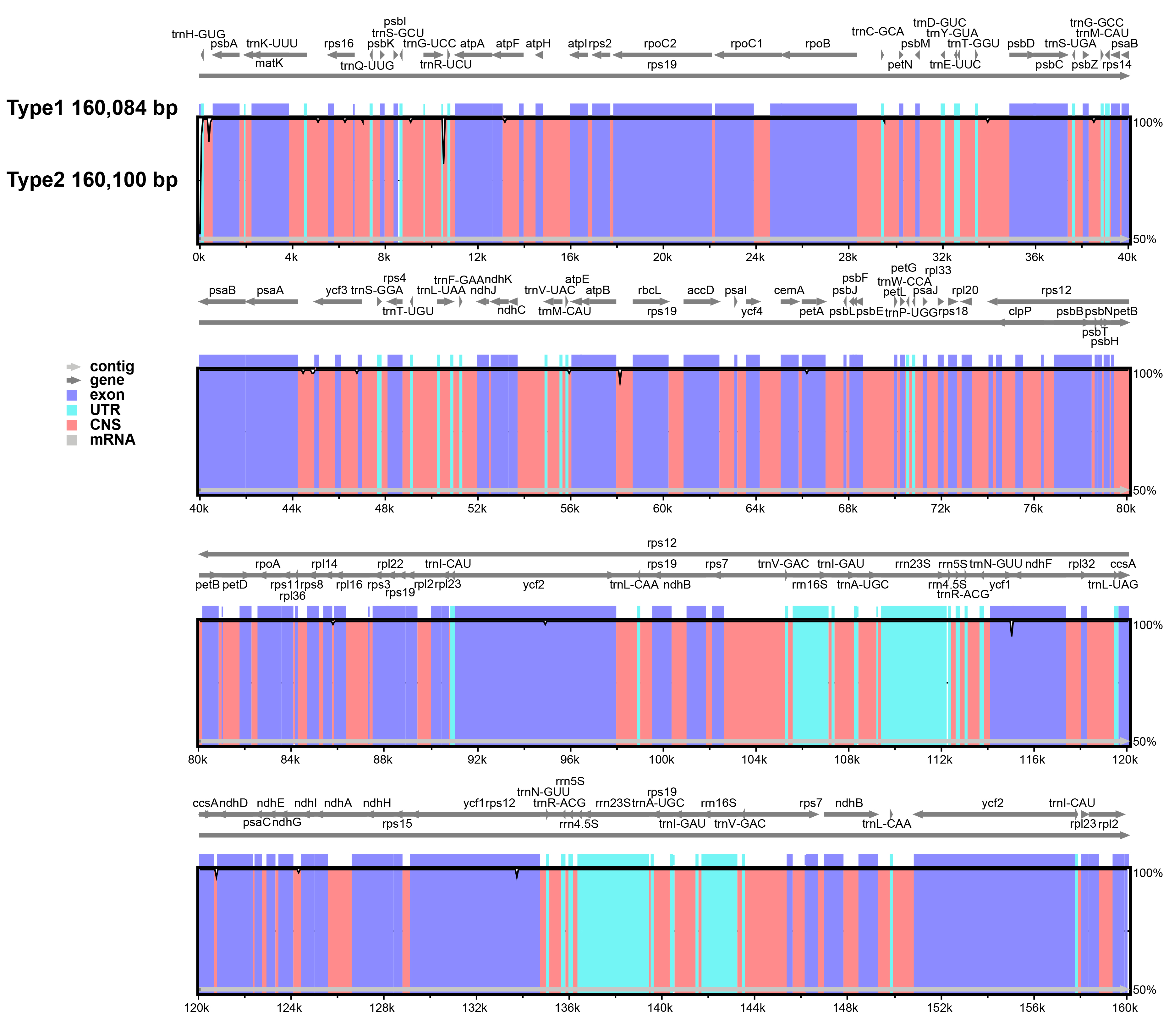


Figure S3 Sequence conservation profiles across three papaya types (mVISTA)
